# Granulocyte Colony Stimulating Factor-Mobilized Peripheral Blood Mononuclear Cells: An Alternative Cellular Source for Chimeric Antigen Receptor Therapy

**DOI:** 10.3390/ijms25115769

**Published:** 2024-05-25

**Authors:** Antonio Ballesteros-Ribelles, Alejandro Millán-López, MDolores Carmona-Luque, Concha Herrera

**Affiliations:** 1 Cell Therapy Group, Maimonides Institute for Biomedical Research, 14004 Córdoba, Spain; antonio.ballesteros@imibic.org (A.B.-R.); alejandro.millan@imibic.org (A.M.-L.); 2 Department of Hematology, Reina Sofia University Hospital, 14004 Córdoba, Spain; 3 Department of Medical and Surgical Sciences, University of Córdoba, 14004 Córdoba, Spain

**Keywords:** G-CSF, mobilization, CAR-T, CAR-NK

## Abstract

Lymphocyte collection by apheresis for CAR-T production usually does not include blood mobilized using granulocyte colony stimulating factor (G-CSF) due to the widespread knowledge that it causes a decrease in the number and functionality of lymphocytes. However, it is used for stem cell transplant, which is a common treatment for hematological malignancies. The growing demand for CAR therapies (CAR-T and NK-CAR), both in research and clinics, makes it necessary to evaluate whether mobilized PBSC products may be potential candidates for use in such therapies. This review collects recent works that experimentally verify the role and functionality of T and NK lymphocytes and the generation of CAR-T from apheresis after G-CSF mobilization. As discussed, T cells do not vary significantly in their phenotype, the ratio of CD4+ and CD8+ remains constant, and the different sub-populations remain stable. In addition, the expansion and proliferation rates are invariant regardless of mobilization with G-CSF as well as the secretion of proinflammatory cytokines and the cytotoxic ability. Therefore, cells mobilized before apheresis are postulated as a new alternative source of T cells for adoptive therapies that will serve to alleviate high demand, increase availability, and take advantage of the substantial number of existing cryopreserved products.

## 1. Introduction

Historically, treatment for hematologic cancers has primarily included chemotherapy, radiotherapy, and hematopoietic stem cell transplantation (HSCT) [1]. The latter is the one that remains a cornerstone in clinical practice for some hematological malignancies. However, with recent advances in both molecular and cellular knowledge and a greater understanding of tumor functionality, numerous alternatives have emerged within the field of targeted immunotherapy. In this area, some therapies have been highlighted, such as monoclonal and bispecific antibodies, conjugated antibodies, checkpoint inhibitors, and (more recently) adoptive cell therapy [2].

Adoptive cell therapy and (more concretely) its development in the form of specific therapy has emerged as a solid alternative for patients with refractory or relapsed hematological cancers [3,4]. Among these, the application of chimeric antigen receptor (CAR) therapy in addressing hematologic malignancies has sparked considerable enthusiasm in recent years. Viewing it through the lens of transfusion medicine, the adoption of CAR-T therapies holds the potential to become a fundamental and enduring treatment not only for hematologic cancers but also for solid-organ malignancies. CAR-T has revolutionized the treatment of hematological malignancies and has achieved unprecedented responses in recent years, especially in B-cell acute lymphocytic leukemia (B-ALL), non-Hodgkin lymphoma (NHL), and multiple myeloma (MM). This presents a noteworthy prospect for the advancement and broadening of therapeutic options. However, these therapies are still far from being perfect and are not exempt from some negative effects and inconveniences that have been the subject of intense study [4]. To alleviate these disadvantages, different generations of CAR therapy have emerged with specific molecular constructions that improve the functionality of the therapy. In addition, due to the unique characteristics of alloreactivity, recognition, persistence, and cytotoxicity presented by natural killer (NK) cells, efforts are being focused on new CAR-NK therapies [5].

In this context, one of the main challenges that clinicians and researchers encounter daily is obtaining both T lymphocytes and NK cells efficiently and on a large scale to be able to perform this therapy. This fact, together with the growing demand for CAR therapy, makes it necessary to find new ways to advance CAR immunotherapy. An important alternative advance would be the search for new sources of collection of these cells to generate CAR products. Typically, these cells are isolated from PBMCs, UCBs, or immortalized cell lines [6,7,8,9]. However, it is common to find cell products from donors or patients previously mobilized using granulocyte colony stimulating factor (G-CSF), since it is the usual procedure for HSCT treatment. PBMCs are the preferred way to obtain them due to the ease of collection and the number of cells obtained; in this article, we review the feasibility of using these G-CSF-mobilized PBMCs to obtain CAR products, both for CAR-T and CAR-NK [10,11].

## 2. Adoptive Cell Therapy (ACT): The CAR Immunotherapy

Adoptive cell therapy (ACT) has emerged as a prominent form of anti-tumor immunotherapy, particularly for patients with refractory or recurrent hematologic malignancies (R/R). This personalized cancer treatment involves the direct administration of immune cells with anticancer properties. The process involves collecting autologous lymphocytes from the patient or allogeneic lymphocytes from healthy donors, usually via an apheresis process. Furthermore, these cells can come from sources such as PBMCs, UCBs, or immortalized lines. Subsequently, these cells are infused in the patient, usually in cases of malignancy that have not responded to conventional treatments [3]. ACT can be classified into specific and non-specific cellular therapies. Non-specific cell therapy includes infusion of various immune cells, such as cytokine-induced killer (CIK) cells, γ/δ T cells, or natural killer (NK) cells. While the main goal is to improve overall immune function, these cells lack specificity against tumor targets, limiting their effectiveness [12,13,14,15].

The non-specific cell therapy terminology is used due to the mechanism of recognition and action that these cells have. For instance, NK cells [16] do not recognize a specific molecule in malignant cells, but they utilize a combination of mechanisms such as the recognition of the loss of HLA-I by killer-cell immunoglobulin-like receptors (KIRs) or the recognition of stress-induced ligands, for example, by activating the NKG2D receptor. In addition to this, the mechanism of action is based on the release of cytotoxic granules such as perforin and granzyme; expressing death receptor ligands such as FasL, which induces apoptosis when it binds to its receptor on the target cell; or binding to antibody-coated cells through the CD16 receptor, triggering antibody-dependent cellular antibodies and releasing cytotoxic granules. In γ/δ T cells [17], the mechanism of action is very similar, including the release of perforin and granzymes, FasL ligand, and CD16 expression. However, these cells can recognize non-classical MHC-I molecules such as MIC-A as well as CD1, which is present in lipid antigens. These non-specific features make these cells critical players in both tumor surveillance and responses to infection. So, by using them in cell therapy, we aim to improve overall immune function but with limited effectiveness due to the lack of specificity against determined tumor molecular targets [12,13,14,15].

In response to the limitations of non-specific cellular therapy, specific cellular therapy has gained attention. Early studies recognized the drawbacks of non-specific approaches and proposed isolating specific cells, engineering them genetically to express certain receptors against tumors, and expanding their numbers in vivo using cytokines and growth factors for further administration to the patient [4]. However, this therapy faces difficulties associated with obtaining, isolating, and expanding these cells. ACT therapies have used both natural cells derived from a host and, more recently, cells genetically modified with antitumor T-cell receptors (TCRs) or chimeric antigen receptors (CARs). This last option is the one that has gained the most attention in recent years [3,4]

The highest development in specific ACT research has been through the use of redirected T cells to recognize antitumor antigens. In the late 1980s, Dr. Zelig Eshhar’s team developed the idea of redirecting T cells from the MD45 murine T-cell line to target antigens in a non-MHC-restricted manner of choice by inserting a newly constructed cell receptor [18]. From this publication, several research studies were developed to generate the prototypes of modern CAR [19]. Nowadays, CAR-T can be defined as T cells that have been genetically modified for the addition of artificial receptors that are capable of specifically recognizing antigens found in the membranes of cancer cells. Briefly, CAR-T cells recognize and bind to specific antigens thanks to the scFV domain in the chimeric antigen receptor [20]. This binding activates the CAR-T cell through intracellular signaling domains, which induces the release of cytotoxic granules such as perforin and granzyme and the secretion of pro-inflammatory cytokines such as IFN-γ; TNF-α and IL-2 exert cytotoxic effects and lead to the recruitment of additional immune cells, respectively, creating an inflammatory environment that helps to eradicate the target cell.

This field of research has continued for many years, and in 2009 it resulted in the first publication that detailed the manufacturing process for CAR-Ts obtained from PBMCs—specifically, non-mobilized frozen patient-derived apheresis products—to treat patients with R/R leukemia [21]. Several years later, in 2012, Davila et al. published the first results of CAR-T cells applied to the treatment of chronic lymphocytic leukemia (CLL), achieving complete remission in three patients [22].

**Figure 1:**
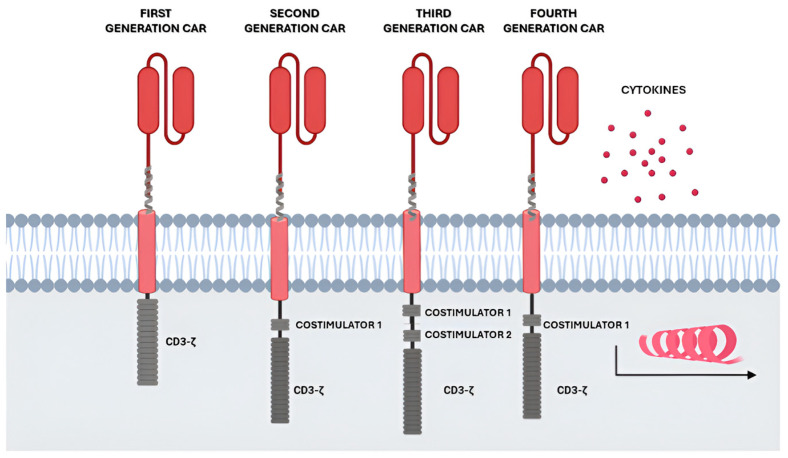
Generations of CAR cells: First-generation CAR cells, equipped with an extracellular antigen-recognizing domain combined with intracellular CD3z accounting for signal transduction. Second-generation CAR cells, equipped with an extracellular antigen-recognizing domain combined with two intracellular domains: CD3z and an additional costimulatory domain like CD28 or 4-1BB. Third-generation CAR cells, equipped with an extracellular antigen-recognizing domain combined with three intracellular domains: CD3z and two additional costimulatory domains. Fourth-generation CAR cells, a diversified group of CAR constructs like cytokine-expressing CAR cells. Created with BioRender.com.

Initially, first-generation CARs consisted simply of a TCR-like construct with a single CD3+ zeta signaling domain [18]. The low persistence in vivo resulted in the second generation of CAR with the incorporation of cytoplasmic domains with costimulatory ability (such as 4-1BB or CD28) into the construct to promote activation, proliferation, and persistence [23]. Subsequently, the research into CAR optimization led to the evolution towards the third generation CAR-T [24], thanks to the addition of two costimulatory molecules such as 4-1BB, CD28, CD27, ICOS, or OX40. A fourth generation, called TRUCKS (t cell redirected for antigen-unrestricted cytokine-initiated killing) [25], has recently been developed. In this fourth-generation CAR, the transgene encoding a second-generation CAR-T is completed with a gene encoding a cytokine such as IL-12 or IL-15 (Figure 1). The purpose of designing cytokine transgenes in TRUCKS is to improve the T-cell activation and antitumor efficacy throughout the release of autocrine and paracrine cytokines by the CAR-T itself, leading to influence over the tumor microenvironment [26]. For example, IL-12 is a powerful amplifier of inflammation, which enhances the cytotoxicity of T and NK cells and induces their release of IFN-γ, affecting the amplification of NK cells and driving the Th1 response. In the case of IL-15, the release of this cytokine suppresses T-cell apoptosis through BCL-2 upregulation, increasing the persistence and antitumor response.

CAR-T therapy has been consolidating as an area of great promise in the treatment of hematologic malignancies since its commercial launch in 2017 with the FDA approval of Kymriah^®^ (tisagenlecleucel) by Novartis [27] and Yescarta^®^ (axicabtagene ciloleucel) by Gilead/KitePharma [28]. Both treatments are based on T cells genetically modified to express CARs targeting CD19 receptors, and they have been indicated for R/R diffuse large B-cell lymphoma (DLBCL) and patients up to 25 years of age with R/R ALL. The beginning of commercial CAR-T therapy brought with it the necessity for safe protocols throughout its administration process. Both commercial therapies are based on autologous cells, so they contain the patient’s T lymphocytes. The CAR-T-cell-manufacturing process begins with the collection of non-mobilized PBMCs from the patient [29], which is accomplished through a leukapheresis process. The collected apheresis products can be processed in several ways depending on the subsequent procedures. For example, in the case of the Novartis product, specific collected cell counts for CD3+ lymphocytes (≥1 × 10^9^ CD3+ cells) are required. Once collected, the apheresis products are sent to a commercial facility where T cells are isolated, activated, genetically modified with a CAR-encoding vector, and expanded before cryopreservation, finally being sent back for infusion into the patient [30].

CD19-targeting CAR T-cell products currently approved by the FDA and EMA include axicabtagene ciloleucel [31], tisagenlecleucel [32], lisocabtagene maraleucel [33], and brexucabtagene autoleucel [34]. Among the CAR-T therapies approved, recent meta-analyses show long-term results of CAR-T treatment [35]. In the case of CAR T-cell therapy targeting CD19 in patients with B-cell lymphoma and CLL, 10 studies with more than 24 months of follow-up were evaluated, establishing overall response rates (ORR) of 44% to 91% and complete remission rates (CR) from 28% to 68%. In the case of therapy for B-ALL with CAR T-targeting CD19, up to 16 studies have been evaluated with a follow-up of more than one year indicating CR rates of more than 80% [36]. Regarding RRMM patients, there are two approved CAR-T products, idecabtagene vicleucel and ciltacabtagene autoleucel, targeting B-cell maturation antigen (BCMA). There are less data from long-term studies with CAR-T-targeting BCMA because these constructs are more recent. Specifically, six studies have been recorded with a follow-up of around one year, reporting ORR rates of 73% to 100% and CR rates of between 33% and 83% [35].

This enormous success reflected in six currently available CAR-T products on the market shows an increase in clinical trials aimed at the treatment of B malignancies such as multiple myeloma, acute myeloid leukemia, and solid tumors. In fact, 1381 CAR-T-cell-based clinical trials were counted on ClinicalTrials.gov on 8 April 2024.

Despite the fact that CAR-T therapy has obtained successful results, it still has several challenges that remain unresolved. Among them, some toxic effects on patients have been described, such as immune-cell-associated neurological syndrome (ICANS) and cytokine release syndrome (CRS) [37]. Moreover, CAR-T therapy relies on autologous peripheral blood-derived and personally engineered cells, which is both time-consuming and costly for patients [38]. Together with additional limitations that involve off-targeted toxicity and lack of effectiveness in solid tumors within the immunosuppressive microenvironment [39], it seems preceptive to optimize CAR products. CAR-T products are improving in different aspects. For example, in the treatment of solid tumors mentioned above, there are numerous clinical trials based on the combination of CAR-T together with inhibitory immuno-checkpoint blockades, such as dual targeting anti-mesothelin/anti-PD-L1 or CTLA-4 CAR-T against malignant mesothelioma [40] and anti-MUC-1 (cell membrane mucin-1) PD-1 knock-out CAR-T cell for adenocarcinomas [41]. This combinational approach can enhance the treatment by blocking PD-1 and decreasing CTLA4, which are critical regulatory pathways for inhibiting T-cell response.

Recently, new CAR cell options have been developed, such as NK-CAR, which could mitigate some CAR-T-related side effects. Similarly to CAR-T cells, CAR-NK cells are NK cells genetically modified to express CARs that recognize a specific antigen uniquely expressed or overexpressed by tumor target cells. It is important to highlight that CAR-NK cells have NK cell characteristics, which means these cells are not only capable of recognizing tumor antigens due to the CAR construction but also eliminating tumors due to the crucial role they play in immune surveillance by identifying and targeting cancerous or virally infected cells that down-regulate HLA class I molecules or express stress markers [5]. Furthermore, the NK cell mechanism of action relies on non-specific stimulatory and inhibitory signals; thus, it could additionally eliminate antigen-negative cancer cells. Besides this, allogenic NK-CAR does not exert GVHD [42], eliminating the necessity of developing different CAR products for each patient, hence it could be a potential candidate for universal off-the-shelf therapy.

GVHD is a severe complication that can occur after HSCT when the donor’s immune cells, particularly T cells, recognize the recipient’s body as foreign and initiate an immune response against the recipient’s tissues [43]. In contrast to T cells, NK cells do not recognize specific antigens presented by MHC on the surface of the host cells, otherwise they are regulated by a balance of activating and inhibitory signals, such as the interaction between KIRs on NK cells and MHC class I molecules on target cells. Thus, since healthy cells usually express normal levels of MHC-I, they are protected from NK-cell-mediated cytotoxicity, reducing the risk of NK cells attacking the host’s normal tissues.

All these advantages, added to the promising preliminary results, are making CAR-NK therapy a promising field in clinical research [44]. There are many published studies regarding NK-CAR applied to preclinical and clinical research, most of them developed using the NK-92 cell line, with the NK derived from umbilical cordon blood (UCB) or hematopoietic pluripotent stem cells (HPSC) [8,45]. Furthermore, there are numerous other CAR-NK clinical trials; concretely, 75 clinical trials were counted from ClinicalTrials.gov on 8 April 2024.

In this scenario, and with the growing demand for CAR therapy, it seems convenient to find new ways to progress in CAR immunotherapy and enhance the clinical trial results. An important alternative approach to optimize this therapy would be the search for new sources of collecting cells to generate large amounts of CAR-T. The design of innovative studies aimed at developing ready-to-use CAR seems essential considering that a single treatment can lead to long-lasting results compared to other therapies, for example, monoclonal antibodies that require prolonged treatment at considerable cost.

## 3. New Cellular Sources for CAR Immunotherapy: G-CSF Mobilized PBSC Products

The development of CAR therapy has introduced a new field of therapeutic possibilities for patients with certain hematologic malignancies; however, the HSCT remains a cornerstone in clinical practice. This treatment is considered a preferred treatment for some high-risk and R/R hematological malignancies. Moreover, with the improved supportive care and increasing acceptance of haploidentical transplantations as an alternative treatment modality, more patients are opting for HSCT as a definite treatment for hematological malignancies. The database, which includes all of the stem cell transplants conducted, includes information on more than 500,000 transplants. Over the last five years, 35,000 new patients have been treated annually. In Europe, more than 400,000 patients treated with HSCT have survived [46].

This therapy aims to infuse hematopoietic stem cells derived from the patients themselves (autologous) or healthy donors (allogeneic). HSCs can be isolated from bone marrow (BM), peripheral blood (PB), or UCB [6,7]. The first successful human HSCT was a bone marrow transplant, performed in 1956 by Dr. E. Donnall Thomas and his research team [47]. The patient was a five-year-old child with leukemia. The procedure involved transferring bone marrow from his healthy identical twin. This landmark achievement laid the foundation for modern-day bone marrow transplantation. In past years, hematopoietic cells were obtained directly from large volumes of bone marrow, aspirated from the iliac crests and using general anesthesia. However, since 1994 and the initial demonstration that peripheral blood stem cells (PBSC) mobilized by cytokines (G-CSF first and more recently plerixafor when needed) could be used as well as BM, the proportion of PB transplants has increased to about 70–95% [48].

Over the past decades, apheresis has become a standard procedure in clinical hematology, providing HSCs for hematopoietic reconstitution after myeloablative chemotherapy. For this reason, and due to the reduced proportion of HSCs in peripheral blood (hovering at 0.04%), G-CSF is routinely utilized before apheresis to mobilize HSCs from BM to PB [46].

Granulocyte colony stimulating factor (G-CSF) is a 19 kDa glycoprotein-containing 175 amino acid residue that functions similarly to a hormone or cytokine [49]. Naturally, the pleiotropic cytokine is produced by activated monocytes, macrophages, endothelial cells, fibroblasts, astrocytes, osteoblasts, and bone marrow cells. Overall, G-CSF binds to G-CSFR (receptor) in monocytes, neutrophils, HSPCs, and endothelial cells, enhancing the bone marrow to produce progenitor cells and release them into the bloodstream. It is noticeable that this molecule not only exerts its function in hematopoietic stem cells but also in different progenitors such as myeloid, erythroid, and megakaryocytic HSPCs [50,51].

The biochemical effects of G-CSF start when it binds to the G-CSFR anchored on the surface of the cells. This fact causes its dimerization, activating a cascade of signals that triggers the activation of three main metabolic pathways [52]. One of these metabolic pathways is JAK/STAT. JAK proteins lead to the phosphorylation of tyrosine residues, activating the STAT3 and STAT5 proteins and inducing their translocation to the nucleus, resulting in an activation of the genes responsible for proliferation, differentiation, and survival. Besides this, the activation of G-CSFR also causes the stimulation of Ras, which will lead to the activation of the MAPK/ERK pathway, and its translocation to the nucleus will activate genes responsible for cell cycle progression. Lastly, activation of G-CSFR will prompt the phosphatydylinositol-3-kinase (PI3K/AKT) pathway, whose phosphorylation will activate AKT, which promotes cell survival and metabolism.

Since G-CSF increases neutrophil mobilization and maturation, it was initially used in clinical practice to prevent and treat neutropenia [53]. However, nowadays its ability to mobilize HSC from the bone marrow [54] has positioned this molecule as the most popular factor for this purpose, becoming the gold standard and changing the paradigm of stem cell transplantation [55]. This strong performance could be related to advantages such as an increase in the number of different peripheral white blood cells such as lymphocytes, monocytes, and (obviously) HSC [56]; the reduced time for the restoration of neutrophils and platelets post-transplant [57]; better safety; and (importantly, according to clinical trials) the fact that normal donors prefer the donation of HSPCs from blood, instead of donation from pelvic marrow, which is more invasive and dangerous [58].

**Figure 2:**
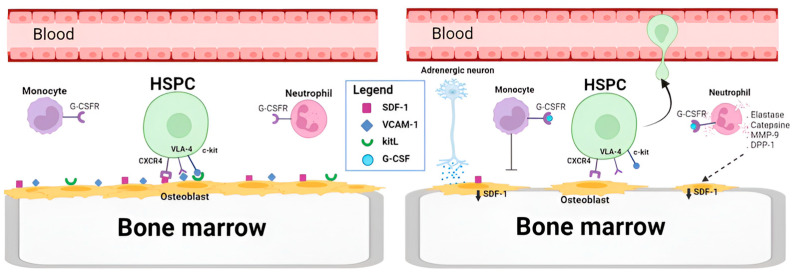
On the left side, cells have not been exposed to G-CSF; therefore, osteoblasts and endothelial cells show on their surface a high expression of cell-adhesion-related molecules, such as SDF-1, VCAM- 1, or kitL. On the right side, cells have been exposed to G-CSF, a molecule recognized by the G-CSFR receptor. This causes neutrophils to release protease enzymes that accumulate in the BM and degrade molecules related to cell adhesion. Furthermore, both the secretion of adrenaline by sympathetic neurons and the secretion of certain factors by macrophages promote a reduction in SDF-1 expression, which leads to the disjunction and mobilization of HSPC cells from the bone marrow. Created with BioRender.com.

Despite the fact that its mechanism of action has been well studied, our knowledge is not complete. There is a hypothesis that envelopes several combinational actors in the mobilization process [59]. In this approach, the adhesion molecules in the BM niche gain special attention (Figure 2). In the normal state, hematopoietic stem cells (CD34+) are situated and retained in the bone marrow through multiple retention axes, such as SCF (Stem Cell Factor)/c-kit; VCAM-1 (Vascular Cell Adhesion molecule-1)/VLA-4; and SDF-1 (Stromal-Derived Factor-1)/Chemokine Receptor-4 (CXCR4) [60]. Studies have confirmed that these interactions are interrupted when G-CSF is used, and this is sufficient for mobilization [61].

In short, this hypothesis suggests that, on one hand, G-CSF prompts neutrophil activation, ending up in a degranulation, releasing some neutrophil’s protease enzymes—such as neutrophil elastase, cathepsin G, dipeptidyl peptidase 1I, and matrix metalloprotease-9 [62]—that can ultimately accumulate in BM and lead to the degradation of cell-adhesion-related molecules such as VCAM-1 [63], SDF-1, and C-kit [61]. Moreover, G-CSF increases CD26, a serine exopeptidase on the surface of endothelial cells that causes internalization and degradation of VE-cadherin, opening endothelial boundaries. G-CSF also triggers erythroblasts to secrete fibroblast growth factor-23 (FGF-23), which counteracts the function of CXCR-4. On the other hand, following G-CSF administration, a reduction in the number of monocytes and macrophages and the suppression of osteoblasts in bone marrow have been observed [64], which could enhance the mobilization process [65,66]. In addition to this, it seems that G-CSF induces sympathetic neurons to release noradrenaline, which promotes the suppression of osteoblast [67] macrophages [68] to release unknown factors that suppress SDF-1 expression on the surface of niche cells.

Overall, G-CSF affects various processes that in combination can disrupt the bone marrow microenvironment, leading to the mobilization of hematopoietic stem cells. These processes include reducing SDF-1 expression, opening endothelial barriers, and counteracting the function of CXCR4 due to the increase of erythroblast-derived fibroblast growth factor-23 (FGF-23) [69].

**Figure 3:**
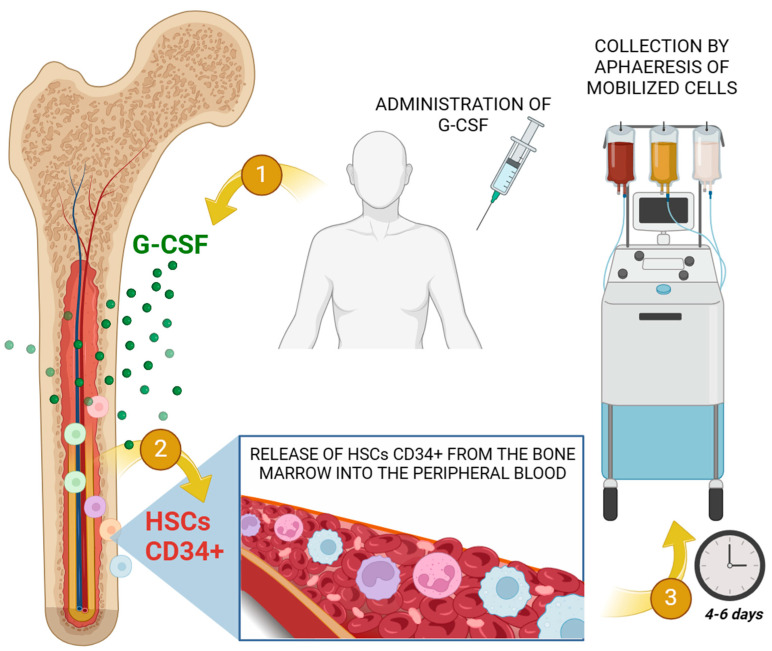
Illustration of the clinical aspects of the apheresis process for the collection of HSCs through the therapeutic administration of G-CSF, showing the process of mobilization of CD34+ cells from the bone marrow to the peripheral blood for subsequent collection. Created with BioRender.com.

Mobilization of HSC from bone marrow via G-CSF is undoubtedly a crucial phase in both auto-HSCT and allo-HSCT. The process encompasses a G-CSF average dosage administration in patients of approximately 5–10 μg/kg for 5–7 days, obtaining around 2 × 10^6^ CD34+ cells per kg weight. Leukapheresis is generally performed on day 5 (Figure 3). It is not compulsory to quantify the amount of CD34+ cells but nowadays CD34+ cell count in mobilized peripheral blood product is the most important parameter of graft quality, due to the fact that it is the only predictor of stable engraftment after auto-HSCT. Finally, HSC will be cryopreserved using dimethyl sulfoxide (DMSO) until infusion [46].

Therefore, it is fairly noticeable that nowadays there are a large number of G-CSF-mobilized and cryopreserved apheresis products both from donors and patients, stored in biobanks for HSCTs main purpose, which could also be used to obtain T lymphocytes and NK cells for the subsequent generation of CAR products.

### 3.1. CAR-T Immunotherapy

Since CAR-T therapy has been proposed as an ultimate procedure after standard cancer treatment, it seems reasonable to think that T lymphocytes for CAR-T therapy could be isolated and expanded from the same cryopreserved product obtained for HSCT, in order to combine both treatments. The paradigmatic case of this potential use would be multiple myeloma or NHL, where many patients undergo CAR-T therapy after relapse following autologous transplantation [70]. In the case of LAL, successful cases have been described with the infusion of allogeneic CAR-T cells from the donor after early relapse following allogeneic HSCT [71,72,73]. In addition to this, this mobilized product could be used to treat the hematopoietic toxicities of autologous CAR-T-cell therapies, which are a major concern, and there are many cases reported that have required the infusion of hematopoietic progenitors as rescue [74,75].

In all these circumstances, it would be beneficial to have a single procedure for apheresis, but all of them require the administration of G-CSF as a mobilization agent, and this growth factor has not been considered for the manufacture of the CAR-T products among the different parameters that have been previously well-determined [4].

Several studies on the functionality of T lymphocytes after mobilization provide reasons not to use this cell source for CAR-T-cell production. In the late 1990s, a series of publications studied mobilized stem cell samples and concluded that G-CSF has a pleiotropic effect in different cell populations, including monocytes which inhibit T-cell proliferation and function [56,76]. First, Young et al. informed the induction of immunosuppressive cells associated with myelopoiesis and stimulated by GM-CSF and IL-3 [77]. Subsequently, Ino K. et al. analyzed the mobilized apheresis product of 21 patients with malignancies. They carried out different cellular inhibition assays and showed that the low-density fraction enriched with CD14+ significantly inhibited the functions of T cells and led to activation-induced apoptosis of these cells [78]. These findings suggest that the augmented monocyte fraction is responsible for the impaired function and inhibition of T-cells after mobilization.

This could be an important reason not to utilize G-CSF-mobilized PBMNC for immunotherapeutic purposes. However, one feasible way to mitigate this T-cell misfunction is to isolate and expand the T-cell CD3+ fraction specifically [79]. Nowadays there are a variety of kits widely used that are designed to enrich solely this CD3+ fraction [80,81]. Studies such as that by Ji et al. demonstrate that G-CSF priming does not change the total number of CD3+ cells in marrow grafts but decreases CD4+ cells and increases CD8+ cells, resulting in a significant reduction in CD4:CD8 ratio [82].

Besides the interactions with other non-lymphocytic cells, several publications have concluded that G-CSF promotes the generation of the Treg phenotype in T cells [83] (which produces IL-10) [84] and transforms growth factor-β, promoting an increase in lymphocyte T helper Th2 differentiation while suppressing Th1 differentiation [85]. This was also supported by other publications in which transcriptomic analysis of G-CSF-mobilized peripheral blood from donors revealed an upregulation of Th2 genes and Tregs and a downregulation of Th17 and Th1 genes [86]. Overall, these findings suggest that G-CSF negatively affects antigen-specific T cells, and T-cell banking before mobilization might be the best option to optimize T-cell production [87], because the efficiency of CAR-T cells that are generated from T cells exposed to G-CSF could be reduced.

In sharp contrast to all these results questioning the functionality of T lymphocytes in PBMNCs obtained after mobilization are the clinical results of thousands of allogeneic transplants of mobilized PB, in which neither graft versus host disease (GVHD) nor graft-versus leukemia (GVL) effects are reduced by mobilization. Moreover, frozen T-lymphocytes from the original mobilized PBMC graft product are used clinically for immunotherapeutic purposes for the generation of functional virus-specific T cells with anti-viral and tumorigenic functions [88,89,90], and more routinely for donor lymphocyte infusions (DLIs) as a treatment for recurrent malignant neoplasms. DLI is capable of eradicating minimal residual disease and rescuing hematological decline, being able to induce lasting remissions [91]. It mainly works well to treat mixed chimerism in which there is a persistent or increasing number of malignant host cells after allo-HSCT, which is a predictor of disease relapse. DLI [92] has the potential to improve the GVL effect, reducing the risk of relapse in patients with mixed chimerism. With the increased use of unrelated donors for hematopoietic cell transplantation, there is renewed interest in the use of large volumes of frozen mobilized apheresis products that could represent a source for DLI as a complementary treatment to prevent or treat the appearance of mixed chimeras.

Considering the benefits previously mentioned and with the knowledge of the clinical success of T lymphocytes after mobilization [88,89,90], researchers have recently opened the debate about its utilization for purposes such as CAR-T-cell production. In fact, it is not unreasonable to think that with the current sample processing and enrichment techniques, one could use a determined volume of the mobilized product to isolate solely the T-cell fraction without altering the functionality of these cells [80]. Some examples using a variety of cytokines and activation protocols for T-cells [93] can obtain different subpopulations. In addition, other techniques such as cell sorting could solve the problems resulting from the effect that other cells such as monocytes have on the suppression of T lymphocytes by choosing the optimal CD3+ subpopulation [94].

Recently, innovative studies have analyzed the effect of the administration of G-CSF to obtain CAR-T cells, such as the study conducted by Cummins et al. [11]. They extensively evaluated this effect through the analysis of cryopreserved apheresis samples derived from four healthy donors, both mobilized with G-CSFrh and unmobilized. The study included the cellular culture where T cells were activated ex vivo using CD3/CD28 beads, transduced with a lentiviral vector, and expanded in a specific cell-culture media enriched with human serum and IL-7/IL-15. Subsequent analysis by flow cytometry, CyTOF, single-cell RNA sequencing (RNA-Seq), and metabolomics by mass spectrometry showed, in a non-significant manner, a higher expression of the CAR construct in non-mobilized cells. Remarkably, no significant differences were found in terms of the CD4/CD8 ratio obtained in both groups, and no differences resulted in terms of degranulation, cytokine production, or in vitro tumor cytotoxicity assays. In vivo analysis of xenografts in an acute myeloid leukemia mouse model showed no statistical differences in mouse body weight, toxicity, or survival in both analyzed groups. Finally, the RNAseq analysis showed a similar expression transcriptomic profile for both groups. The research indicates that the antitumor efficacy and in vivo toxicity of these products are comparable, with no significant differences observed in the product exposed to G-CSF, as determined through multi-omics analyses.

In another recent comprehensive study, Canesin et al. [95] determined the impact on the apheresis product arising from mobilization (or lack of) with G-CSF and plerixafor from 30 healthy donors, by assessing immune cell composition, T-cell phenotype, and T-cell functionality in controlling AML tumor growth following anti-CD33 CAR transduction. The resulting in vivo immunophenotypic analysis yielded remarkably interesting results showing that mobilization decreases the overall percentage of CD3+ T cells but increases naive (CD45RA+/CCR7+) T cells and decreases the T-cell population of effector memory (CD45RA-/CCR7-) and central memory (CD45RA-/CCR7+). In vitro functional cytotoxic assays demonstrated that mobilized-antiCD33-CAR T cells were as effective as non-mobilized-antiCD33-CAR T cells in killing CD33+ AML cells.

In the domain of multiple myeloma treatment, Battram et al. [96] proposed utilizing G-CSF-mobilized leukapheresis products to obtain CAR-T cells targeting BCMA. Their study revealed the minimal impact of G-CSF on T-cell phenotype, both in vitro and in patients. Pre-treatment with G-CSF did not affect T-cell survival or apoptosis during culturing, activation, transduction, or expansion. CAR T production was unaffected, with no impact on cell growth, differentiation, or anti-tumor killing capacity. Exhaustion markers like PD-1, LAG-3, TIM-3, and TIGIT showed no significant increase with G-CSF, except for reduced TIM-3 expression in CD8+ cells, this being the only significant difference indicating less exhaustion.

In PBMCs obtained from MM patients through G-CSF-mobilized and non-mobilized apheresis [96], no significant differences existed in the CD4:CD8 ratio or Treg population. G-CSF reduced TSCM cell frequency, specifically CD8+ TSCM cells, without affecting other memory and effector T-cell populations. Although CAR transduction is statistically lower in mobilized cells, the high expansion rates and increased T-cell numbers compensate for this reduction. In vivo functionality of CAR-T cells, assessed in a mouse model with MM xenografts, shows similar disease development in CAR-T-treated mice from mobilized and non-mobilized cells. In summary, the study supports the feasibility of using CAR-T cells obtained from G-CSF-mobilized leukapheresis products for treating multiple myeloma. Despite a reduction in CAR transduction, the high cell expansion compensates for this effect, suggesting that these CAR-T cells could be an effective therapeutic option for multiple myeloma.

Another recent retrospective study by Künkele et al. [10] has been carried out using cryopreserved PBMC-mobilized apheresis products derived from eight patients diagnosed with neuroblastoma. Samples were obtained early in the treatment protocol. To activate and expand the T cells, a monocyte depletion of thawed PBSC units was required due to the outgrowth of monocytes. Furthermore, the CAR transduction efficiency of CD4+ and CD8+ mobilized products to generate the CAR-T cell product ranged from 65 to 75%. Besides this, flow cytometric analysis showed that cryopreserved G-CSF-stimulated apheresis products contained sufficient numbers of CD4+ and CD8+ T-cell precursors with a naïve and central memory phenotype that showed increased replicating potential and a high capacity to generate large numbers of effector T cells after tumor stimulation.

Lastly, other research works related to human γδ T cells, such as the study performed by Otto et al. [97], have shown that the PBMCs G-CSF mobilized and derived from healthy donors succeeded in retaining their cytotoxicity and in the production of a variety of cytokines. These cells produced considerable amounts of IL-6, IFNγ, TNFα, and GM-CSF, cytokines that are important in mediating cytotoxicity and supporting inflammation. Other cytokines released were IL-4, IL-5, IL-8, IL-13, G-CSF, MCP-1, and MIP-1β, relevant factors that help attract other effector cells. As for the cell phenotype, significant differences were observed in the phenotype of isolated γδ-T cells compared to non-mobilized γδ-peripheral donor T cells. There was increased expression of CD8, CD56, CD28, and CD11b/CD18 (MAC-1) in these isolated cells. This evidence suggests that these cells would have the ability to modulate the immune responses and play a significant role in adoptive immunotherapy in the same way the non-mobilized cells would do.

Overall, these experimental publications have some things in common. As Battram et al. mentioned, the way they proceed is different from the late 90′s experimental approaches due to the reduced exposure time of the G-CSF and the starting material for the ex vivo culture of the CAR-T. This source of starting material should be highly purified T cells and not the whole PBMC fraction [96]. This would lead to a major advantage thanks to the removal of T-cell-inhibitory cells such as the monocyte CD14+. This particular principle is included in the Advanced Therapy Medicinal Products (ATMPs) guidelines of CAR-T therapy. The guidance [98] for ATMPs indicates that the CAR-T-cell-manufacturing process usually requires 12 days, and starts, in short, with the isolation of T cells from the leukapheresis product of a patient, followed by activation and genetic modification of the cells to express the respective CAR. Thus, taking advantage of the fact that cells must be routinely isolated, cultured, usually sorted by cell phenotype, and treated appropriately, it seems reasonable to assume that prior mobilization of G-CSF would not significantly affect the protocol, with these lymphocytes being fully functional, as explained throughout this section.

Besides this, as we mentioned previously, G-CSF mobilization products may not be the first indicated option to obtain T-cell fraction; however, it can be seen that cryopreserved, stored, and mobilized PBMCs could perform as well as non-mobilized ones in terms of CAR-T production.

### 3.2. CAR-NK Immunotherapy

One of the main limitations in the development of CAR-NK is obtaining NK cells in massive quantities, since these cells are found in a lower proportion (from 5% to 15% of blood circulating lymphocytes) than T or B lymphocytes under normal circumstances [45]. Source and clinical scale-up of NK cells with long-lasting cytotoxicity activity are the main challenges, and the strategies differ depending on whether the sample is freshly isolated, activated, or in vitro expanded because of the variety of phenotypic and functional differences found. Nowadays, the principal source of obtaining NK-CAR for clinical trials is the NK92 [99,100] cell line, due to the lack of variability that this cell line offers, together with its unlimited proliferation ability and modest cost. Nevertheless, there are some drawbacks, including the lack of CD16 expression and a higher tumorigenicity risk because it is a tumoral cell line [101]. To circumvent these problems, it seems necessary to find new tools to broaden the source of these cells in order to obtain an “off-the-shelf” therapy. Other NK cell sources have been studied, for instance, those isolated from PBMCs, UCBs, CD34+ hematopoietic progenitor cells [8], and induced pluripotent stem cells (iPSCs) [9].

One source in recent use is PBMC-derived CAR-NK. There are some fairly strong advantages, such as the uncomplicated method of collection and the lack of GVHD that facilitates isolation between matched and mismatched HLA donors [102]. Besides this, the cells collected using isolation kits such as CD3+ depletion and CD56+ enrichment are CD56dimCD16+, which is a more cytotoxic and mature phenotype than the NK92 line [103]. Furthermore, even though they have a reduced capacity for proliferation when compared to other phenotypes, with proper isolation and cell culture these cells can expand quite well. Another option is to obtain NK from UCB in the same manner, although this leads to reduced numbers of UCB NK due to the limited volume of UCB units [104].

Since PBMCs seem to be a good option for obtaining NK cells for CAR manufacture, it is not far-fetched to think that, similarly to CAR-T, cryopreserved G-CSF-mobilized apheresis products could be used to obtain these cells. Nevertheless, as with CAR-T, the use of apheresis products mobilized using G-CSF is not a common protocol to obtain the NK cell isolate, since the apheresis mobilization process does not aim to increase the number of lymphocytes or NK cells [105]. On the one hand, Clausen et al. [106] first approached this question elegantly and found a strong impairment on NK cell in vivo expansion and proliferative capacity after G-CSF, with monocyte depletion also insufficient. Subsequently, they found that the interaction between CD34+ progenitor cells, together with T cells, is essential for the suppressive effect on NK cell expansion, while purified NK cells from mobilized products were unimpaired [107].

Recently, some studies have also indicated that the mobilization process can negatively affect the functionality of NK cells [108], due to the over-proliferation of polymorphonuclear myeloid-derived suppressor cell (PMN-MDSC) subpopulations [109] and the inhibition of IFN-Y secretion by them [110].

However, there is a debate (well-reviewed by Gazitt Y. [111]) in which different publications related to cell populations from mobilized PBMCs such as CD34+ cells, T-cells, NK cells, and dendritic cells were analyzed, including his own experimental results. The paper highlights that there are other studies that do not find differences between the mobilized and non-mobilized products in terms of NK, noticing a lack of consensus. In addition, a great heterogeneity is observed in terms of NK cell mobilization between patients, perceiving that those patients with relatively low NK cell or poor NK activity before mobilization had poor PBSC collection post-mobilization in terms of quantity and functionality. Thus, it is pointed out that these variables, together with the small number of patients included in some of the experimental publications analyzed, should be taken into consideration and the mobilization process itself could not be an issue.

A recent publication by Xiong et al. [108] showed significant differences between non-mobilized and mobilization-derived PBSC NK. In this case, the mobilized NK fraction showed a CD56bright+ CD16- phenotype population, suggesting that G-CSF favors the accumulation of less mature NK cell subsets. Besides this, they observed a decreased expansion rate in mobilized NK compared to non-mobilized NK. However, when IL-15 is added to the culture, a progression and restoration in cytokine secretion profile in vitro can be observed, suggesting that these cells can experience a maturation. Notwithstanding the potential perception of this aspect as a drawback, expeditious dismissal of its optimization is unwarranted. It is imperative to recognize that presently, one of the preeminent sources of NK-CAR resides in NK-92, a cell line characterized by CD56+CD16-, which exhibits low cytotoxicity and in vivo persistence. Besides this, as we mentioned before, the manufacture of CAR requires an optimal culture of the highly purified cells, thus it is reasonable to think that to design a CAR NK cell product, a lower maturation stage and an ex vivo culture modulation through cytokines should be a promising alternative source to certain NK-CAR phenotypes [112,113].

Otherwise, as NK cells originate from CD34+ hematopoietic stem cells, one feasible way to obtain NK cells is to focus on the source of this population, which encompasses bone marrow or umbilical cord blood. As shown by Oberoi et al. [114] in their study, the effect of G-CSF can also be used to obtain the mobilized CD34+ population from peripheral blood in massive quantities and in a simple manner, to subsequently differentiate into NK cells. These cells are then expanded and differentiated into mature NK cells using a cocktail of cytokines in a culture system. There have been many advances in the way of culturing, expanding, and differentiating functional NKs, for example, by co-culture with K562 feeder cells [115] that co-express the 4-1BB ligand and membrane-anchored IL-15 and IL-21, giving excellent results and solving the problem of variability between donors. The resulting CD56+CD3− NK cells are mostly similar to PB NK cells, express NK cell-activating receptors, and exhibit potent cytotoxicity against leukemic cells in vitro and in vivo.

Some studies, such as the one published by Patel et al. [105], have designed and evaluated a CAR-NK development protocol using NK derived from PBSC mobilized with G-CSF. The results derived from these studies achieved the differentiation of NK cells in the culture using cytokines such as IL-7 and IL-15, obtaining cell proliferation and expansion rates equivalent to those obtained from UCB, with a CD56+/CD16+/CD94+ phenotype showed by 10–40% of the CD56+ cells. They concluded that large-scale generation of CAR-NK products from PBSC sources is feasible and compatible with good manufacturing practice (GMP), which is mandatory for ATMP products; it is defined as potent products manufactured safely according to standardized methods under controlled, reproducible, and auditable conditions [46].

Finally, Zhu et al. [116] have achieved remarkable success in invariant natural killer (iNKT) cell generation, through TCR genetic engineering of peripheral CD34+ HSCs samples mobilized by G-CSF. iNKT are immune system cells with a strong potential to fight cancer. However, their clinical use has been limited due to its scarcity in cancer patients. In their study, they developed a proof of concept for cell therapy using HSC-iNKT, to provide sustained therapeutic levels of iNKT throughout treatment. Using a mouse model with human hematopoietic stem cell grafts and human T-cell-receptor genetic engineering for iNKT, these researchers demonstrated the efficient and long-term generation of HSC-iNKT cells in vivo.

These HSC-iNKT cells showed similarity to natural human iNKT cells and exhibited multiple mechanisms to attack tumor cells, effectively suppressing tumor growth in mouse models with human tumor xenografts. In addition, preclinical safety studies that were performed revealed no toxicity or tumor formation associated with HSC-iNKT cell therapy. Mobilized HSC sources present a greater number of iNKT than non-mobilized samples, emphasizing its potential and safety in hematologic malignancy cell therapy.

Overall, similarly to CAR-T immunotherapy, as CAR-NK manufacture is yet to be explored, it seems necessary to increase the experimental work related to NK cells and to exploit other potential sources available.

## 4. Conclusions

In the context of manufacturing CAR immunotherapeutic products, mobilization with G-CSF, performed to increase the number of precursor hematopoietic cells in the peripheral blood before cell collection, does not adversely affect the manufacture of CAR-engineered cells for several reasons.

Although G-CSF mobilization can increase the number of T cells or NK cells in the peripheral blood, it usually preserves the functionality and essential features of these cells. This includes the ability of these cells to be genetically modified and subsequently expanded in the CAR-T or CAR-NK manufacturing process. T cells and NK cells, mobilized with G-CSF, are compatible with the collection, genetic modification, and expansion processes carried out in the laboratory to produce CAR-T or CAR-NK. Mobilization with G-CSF does not adversely affect the ability of these cells to be modified with CAR, or their ability to proliferate in sufficient quantities.

Specific studies and analyses have evaluated the impact of G-CSF on these cells used in cell therapy, and the results have suggested that cells mobilized by G-CSF are suitable for CAR manufacturing, with comparable results in terms of efficiency and safety. It is important to note that although mobilization with G-CSF is well tolerated, there may be variations among patients, and it is essential to conduct specific evaluations in each case to ensure the efficacy and safety of CAR therapy.

This review highlights the benefits and the potential feasibility of creating and designing cellular immunotherapies that complement hematopoietic stem cell transplantation. The focus is on using the same mobilized collection for both purposes and finding a method to utilize previously cryopreserved mobilized units. This approach not only streamlines the process by using the same collection for multiple therapeutic purposes but also considers the potential advantages associated with the use of historically cryopreserved T cells, particularly in terms of reduced exposure to chemotherapy.
